# Longitudinally Extensive Transverse Myelitis: A Sub-Saharan Conundrum

**DOI:** 10.1155/2020/8879121

**Published:** 2020-12-09

**Authors:** Antonina Obayo, Sylvia Mbugua, Sayed K. Ali

**Affiliations:** ^1^Department of Medicine, Aga Khan University, Nairobi, Kenya; ^2^Neurology, Department of Medicine, Aga Khan University, Nairobi, Kenya; ^3^Internal Medicine, Department of Medicine, Aga Khan University, Nairobi, Kenya

## Abstract

Spinal cord schistosomiasis is a rare, underdiagnosed manifestation of schistosomiasis. We present the case of a 36-year-old male who presented to our institution with a one-week history of low back pain with rapidly progressive lower limb weakness, loss of sensation, and flaccid paraparesis. An MRI of the spine showed a longitudinally extensive transverse myelitis from T6 to L1, with enhancement at the cauda equina region. Further review of the images and serological tests eventually led to diagnosis of spinal schistosomiasis. He was treated with praziquantel and high-dose steroids, with minimal improvement in his symptoms.

## 1. Introduction

According to the World Health Organization, in 2018, 229 million individuals were estimated to be suffering from schistosomiasis. Schistosomiasis is endemic in 78 countries worldwide. In Kenya, approximately 3 million individuals require preventive chemotherapy for schistosomiasis annually [1]. Schistosomiasis accounts for 1 to 4 percent of spinal cord lesions in sub-Saharan Africa. The prevalence of oviposition in the spinal cord varies among studies, ranging from 0.3% to 13% [2]. The clinical presentation of spinal cord schistosomiasis (SCS) is heterogeneous but often presents with low back pain and lower limb weakness sometimes affecting ambulation. Other symptoms include bladder dysfunction, lower limb paraesthesia, hypoesthesia or anaesthesia, deep tendon reflex abnormalities (hyper-, hypo-, or areflexia), constipation, and sexual impotence [2]. SCS usually presents acutely or subacutely and is classified into three clinical forms: medullary with a predominance of spinal cord involvement, myeloradicular, with mainly spinal cord and nerve root involvement, and conus-cauda equina syndrome, with predominantly conus or cauda equina involvement [2–4]. The diagnosis of SCS is based upon the presentation of neurological symptoms resulting from lesions of the spinal cord, the demonstration of schistosomal infection using microscopy and serological techniques, and the exclusion of other causes of myelopathy [4, 5]. Treatment usually includes antischistosomal drugs with corticosteroids [2, 4–6].

## 2. Case

A 36-year-old male from central region of Kenya presented to our institution with a 3-day history of back pain, followed by itching and numbness of the feet and ankles. On presentation, his symptoms had progressed to weakness of the lower limbs with inability to walk and he had developed urinary incontinence. He denied speech and swallowing difficulties and reported no weakness of the upper limbs. The patient reported no history of fevers, blurred vision, loss of consciousness, or seizure. He also denied a history of trauma, use of blood thinners, use of illicit drugs (including tobacco and alcohol use), a history of exposure to tuberculosis, or ingestion of raw milk. He did report swimming in a nearby pond for many years. His family history was unremarkable, and he was an only child.

On examination, he had normal vital signs and was conscious and alert. A neurological exam revealed normal cranial nerves. Visual field was also preserved. Examination of both his upper limbs was normal. He had tenderness of the lower back, power grade 4/5 on hip flexors and extensors bilaterally, grade 4/5 and 2/5 on the right and left knee extensors, respectively, and power grade 3/5 and 1/5 on the right and left knee flexors, respectively, and dorsiflexion was grade 1/5 bilaterally. A knee jerk reflex was present on his right lower extremity but reduced on the left. Ankle reflexes were absent bilaterally. Sensory level was present at L2.

His complete blood count and a comprehensive metabolic panel were within normal limits. A lumbar puncture revealed a protein count of 0.97 g/l (0.15–0.45 g/l), with a pleocytosis of 35 mm^3^(normal <5). A differential count showed 30% of segmented neutrophils and 70% of lymphocytes. Monocytes, eosinophils, and basophils were absent. The glucose level was normal. Cultures of the cerebrospinal fluids, including for tuberculosis, were negative. Serological test for HIV, herpes virus, and syphilis was also negative. An autoimmune screen including ANA, anti-ds DNA, and ENA was negative. A vasculitis screen and serum angiotensin-converting enzyme were also reported as normal.

His thyroid stimulating hormone, vitamin B12, and folate level were all within normal limits.

**Figure 1:**
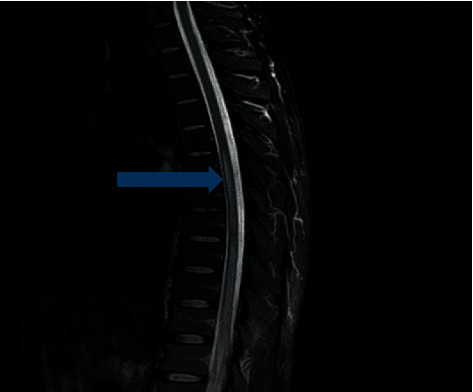
T2 lumbar sagittal, abnormal high signal at the cauda equina.

**Figure 2:**
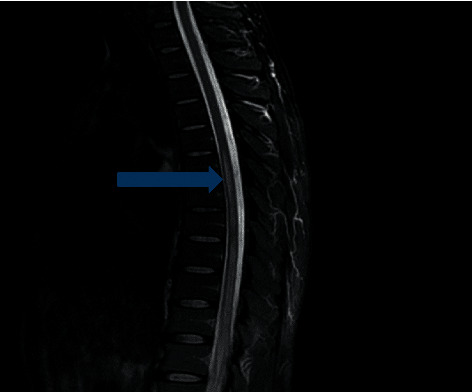
T2 sagittal hyperintense intramedullary signal from T6 to conus medullaris.

**Figure 3:**
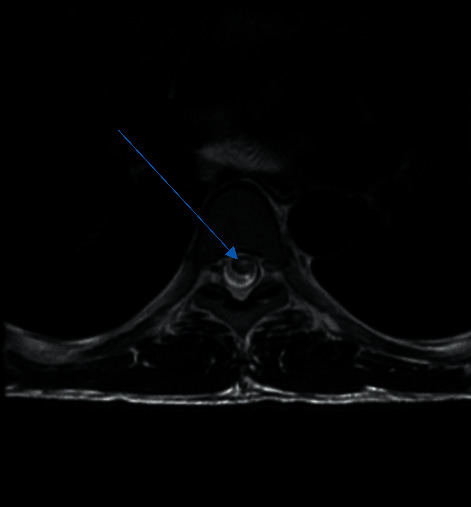
T2 sagittal and axial hyperintense lesion.

An MRI of the spine showed longitudinally extensive transverse myelitis (T2 hyper intense intramedullary signal spanning T6-L1 with enhancement) (Figures 1–3).

Based on his MRI results and clinical symptoms, neuromyelitis optica (NMO) was high on the differential diagnosis. He was initiated on high-dose steroids, methylprednisone one gram intravenously for 5 days. However, his neurological status continued to decline. Five sessions of therapeutic plasma exchange were also attempted with no improvement in his symptoms. Serum and CSF neuromyelitis antibodies and oligoclonal bands as well as serum MOG antibodies (myelin oligodendrocyte glycoprotein) were later reported as negative.

A few days into his hospitalization, his Schistosoma antibody (IgG) titer was reported at at 1 : 80. A subsequent liver ultrasound was reported as normal. He was immediately started on praziquantel at 60 mg/kg/dose and high-dose steroids with minimal improvement in his overall symptoms. He was discharged home with recommendations for home care and aggressive physical therapy.

## 3. Discussion

*Schistosoma mansoni* and *Schistosoma haematobium* are the most common causes of spinal schistosomiasis [2]. Spinal schistosomiasis usually presents as symptoms of spinal cord compression, transverse myelopathy, and painful lumbosacral radiculopathy with low back pain or cauda equina or conus medullaris syndrome [4, 6–9].

Infections like tuberculosis or herpes viruses can present with conus lesions. Inflammatory, vascular, and demyelinating diseases such as multiple sclerosis and neuromyelitis optica can also present in a similar fashion, mostly affecting the cervical and mid-thoracic spine. Spinal cord schistosomiasis differs from the other conditions in that it predominantly affects the lower spinal cord.

In our patient, the diagnosis of spinal schistosomiasis was based on the prevalence of Schistosoma in Kenya, his neurological findings, imaging results, Schistosoma titers, and exclusion of other causes of spinal cord diseases [10]. Even though there was no evidence of hepatosplenic schistosomiasis nor any evidence of schistosomal eggs in urine and faecal samples, this is usually not sufficient to rule out schistosomiasis [2, 10].

Cerebrospinal fluid examination usually shows slight-to-moderate increases in both total protein concentration and lymphocytic count. Eosinophils can present in the CSF in about 50% of patients. A prospective study of 63 patients with schistosomal myeloradiculopathy done in Brazil revealed high total protein concentration in CSF and or pleocytosis in the great majority (95.2%) of the cases [3]. A little more than half (57.4%) of the 63 cases presented with eosinophils in the CSF. In our patient, CSF analysis reflected an inflammatory pattern characterized by increase in the total protein concentration and pleocytosis. Eosinophils were not found.

Enlargement of the lower thoracic spinal cord, swelling of the conus medullaris, and thickening of spinal roots that form the cauda equina are some of the findings on an MRI. Furthermore, high-intensity signal on T2-weighted sequence and heterogeneous enhancement of gadolinium contrast on T1-weighted sequence can be observed in the acute spinal neuroschistosomiasis [2, 5, 10].

A definitive diagnosis of spinal schistosomiasis would require biopsy of the lesion which is associated with potential serious consequences like worsening of neurological signs and symptoms [2]. Due to the risks involved, our patient opted out of a biopsy.

No specific clinical picture is pathognomonic of spinal schistosomiasis. However, conus and cauda syndrome with intramedullary lesion in an endemic area should raise the suspicion of schistosomiasis [2, 3, 11].

Treatment with praziquantel and steroids is associated with clinical improvement, even when started late in the course of the disease [5]. Significant improvement was observed in lumbar and lower limb pain, urinary and intestinal dysfunction, lower limb strength, and ambulation within the first two months of therapy [5]. Some studies have shown that early treatment is better than late treatment [3]. Once treatment is initiated, the outcome of SCS is good [3, 5, 10].

Management of SCS is multidisciplinary and involves the participation of various professionals including nurses, physiotherapists, general clinicians, neurologists, psychologists, and occupational therapists. Physiotherapy remains key for patients with impaired motor functions and must be maintained even after the termination of corticosteroid therapy [10]. In our patient, a follow-up visit 3 months later showed improved bilateral plantar and dorsiflexion of his lower extremities with power grade 2/5 with plantar and dorsiflexion. However, he had power grade 2/5 on hip flexors and extensors bilaterally, grade 2/5 on the bilateral knee extensors respectively, and power grade 1/5 on the bilateral knee flexors.

## 4. Conclusion

Spinal schistosomiasis is a rare manifestation of schistosomiasis. Definitive diagnosis of spinal cord schistosomiasis is made by detection of the eggs in a spinal cord biopsy or at autopsy. However, a presumptive diagnosis can be made from involvement of the lower spinal cord especially the conus medullaris and cauda equina, suggestive clinical picture, and history or evidence of active schistosomiasis. Exclusion of other conditions is also essential for diagnosis.

## Data Availability

No data were used to support the study.
